# Downregulation of phosphorylated MKK4 is associated with a poor prognosis in colorectal cancer patients

**DOI:** 10.18632/oncotarget.16128

**Published:** 2017-03-11

**Authors:** Pu-Ning Wang, Jun Huang, Ying-Hua Duan, Jia-Min Zhou, Pin-Zhu Huang, Xin-Juan Fan, Yan Huang, Lei Wang, Huan-Liang Liu, Jian-Ping Wang, Mei-Jin Huang

**Affiliations:** ^1^ Department of Colorectal Surgery, The 6th Affiliated Hospital, Sun Yat-sen University, Guangzhou, China; ^2^ Department of Traditional Chinese Medicine, The 1st Affiliated Hospital, Sun Yat-sen University, Guangzhou, China; ^3^ Department of Pathology, The 6th Affiliated Hospital, Sun Yat-sen University, Guangzhou, China; ^4^ Gastrointestinal Diseases Research Institute of Guangdong Province, The 6th Affiliated Hospital, Sun Yat-sen University, Guangzhou, China

**Keywords:** phosphorylated mitogen-activated protein kinase kinase 4, colorectal cancer, metastasis

## Abstract

Mitogen-activated protein kinase kinase 4 (MKK4) is a key mediator of Jun N-terminal kinase signaling and influences malignant metastasis. Here, we used immunohistochemistry to assess phosphorylated MMK4 (pMKK4) levels and examine their association with the clinicopathological features of a pilot set of patient samples consisting of normal colonic mucosa (NCM), colorectal adenoma (CA), and colorectal cancer (CRC) tissues. pMKK4 levels were also assessed in a validation set of CRC cases with accompanying follow-up data to confirm their clinicopathological and prognostic significance. pMKK4 levels, which were high in 79.17% of NCM samples, were downregulated in 33.33% of CA and 63.54% of CRC samples. pMKK4 downregulation was associated with metastasis, especially to the liver. In the validation set, pMKK4 downregulation was associated with increases in invasive depth, lymph node metastasis, distant metastasis, and TNM stage. Univariate analysis indicated that pMKK4 score, tumor differentiation, and TNM stage were correlated with disease-free survival and overall survival. Multivariate analysis indicated that decreased pMKK4 expression was an independent risk factor for disease-free survival in CRC patients. These results suggest that CRC patients with low pMKK4 immunochemistry scores should be monitored carefully for early detection of possible recurrences, especially liver metastasis.

## INTRODUCTION

Mitogen-activated protein kinase kinase 4 (MKK4), a member of the mitogen-activated protein kinase (MAPK) family, is involved in the stress-activated protein kinase (SAPK) pathway and is phosphorylated and activated by mitogen-activated protein kinase kinase 1 (MKK1) [1]. Phosphorylated MKK4 (pMKK4) in turn phosphorylates and activates JNKs and p38 in response to cellular stresses and pro-inflammatory cytokines. However, phosphorylation at serine 80 inactivates MKK4 and inhibits its effects on JNK/p38 signaling [2]. In contrast, dual phosphorylation at serine 257 (S257) and threonine 261 (T261) might further enhance MKK4-induced JNK/p38 signaling [3]. pMKK4 thus affects a variety of biological processes, including apoptosis, cell differentiation, and gene transcription.

Although positional cloning coupled with *in vivo* m1etastasis assays suggested that MKK4 is a metastasis suppressor gene [4], the role of MKK4 in tumor progression and metastasis requires further investigation. MKK4 has also been implicated in apoptosis and neoplastic transformation. Genetic inactivation of the MKK4 gene on chromosome 17p has been reported in pancreatic, biliary, and breast carcinomas, suggesting that MKK4 acts as a tumor suppressor gene in these tumors. Furthermore, progressive loss of MKK4 expression has been reported in prostate and ovarian cancers [3, 5, 6]. Downregulation of MKK4 induced the development of a more aggressive cancer phenotype that is more prone to invasion, metastasis, and chemo-resistance [7]. However, in another report, MKK4 protein expression increased in invasive gastric cancer [8]. A recent study also identified MKK4 as a putative tumor marker with possible prognostic value in gastric cancer [9].

The effects of MKK4 in colorectal tumorigenesis have not been well studied. Here, we investigated the role of phosphorylated MKK4 (pMKK4), the activated form of MKK4, in colorectal cancer (CRC). To investigate whether dual phosphorylation of pMKK4 at S257/ T261 is involved in CRC progression and metastasis, we used tissue microarrays to examine pMKK4 expression in a pilot set of normal colonic mucosa (NCM), colorectal adenomas (CA), and CRC samples with different TNM stages. We then examined pMKK4 expression in a validation set containing additional CRC specimens and accompanying long-term follow-up data to explore the clinicopathological and prognostic significance of pMKK4's effects on CRC progression.

## RESULTS

### Clinicopathological characteristics

Clinicopathological characteristics for the 144 patients (M:F = 77:67, 60.5 ± 13.8 years) in the pilot set are shown in Table 1. 438 additional CRC patients (M:F = 230:208, 61.0 ± 14.2 years) were enrolled in the validation set; the follow-up period for these patients was 102.0 ± 45.2 (6.0–168.0) months (Table 2), and their clinicopathological characteristics are shown in Table 3. There were no differences observed in age and gender among patients with high, moderate, weak, and negative pMKK4 scores (Table 4).

**Table 1:** Clinicopathological features of NCM (normal colonic mucosa), CA (colonic adenoma), and CRC (colorectal cancer)

	NCM	CA	CRC			
			Stage I	Stage II	Stage III	Stage IV
Total	24	24	16	23	43	14
Gender						
Male	15	17	11	10	20	10
Female	9	7	5	13	23	4
Age (years)						
Mean	56.7	63.6	62.5	58.0	57.3	58.5
Range	44˜72	41˜82	33˜86	34˜78	18˜80	21˜86
Differentiation						
Well	—	—	6	8	5	3
Moderate	—	—	10	15	29	9
Poor	—	—	0	0	9	2
T Stage						
T1	—	—	4	0	2	1
T2	—	—	12	0	5	0
T3	—	—	0	20	27	10
T4	—	—	0	3	9	3
N Stage						
N0	—	—	16	23	0	4
N1	—	—	0	0	30	5
N2	—	—	0	0	13	5

**Table 2:** Clinicopathological characteristics of the further validation-set of colorectal

Characteristics	*N* = 438
Age (years)	
< 60	201 (45.9%)
≥ 60	237 (54.1%)
Gender	
Male	230 (52.5%)
Female	208 (47.5%)
Tumor Differentiation	
Poor	58 (13.2%)
Moderate	343 (78.3%)
Well	37 (8.4%)
LN metastasis	
Negative	244 (55.7%)
Positive	194 (44.3%)
Distant metastasis	
Negative	311 (71.0%)
Positive	127 (29.0%)
Liver metastasis	
Negative	321 (73.1%)
Positive	117 (26.9%)
TNM Stage	
I	62 (14.2%)
II	169 (38.6%)
III	161 (36.8%)
IV	46 (10.5%)
pMKK4 score	
Low (–/+)	320 (73.1%)
High (++/+++)	118 (26.9%)

(*N* = 438).

**Table 3:** pMKK4 score in NCM (normal colonic mucosa), CA (colonic adenoma) and CRC (colorectal cancer)

Variables	pMKK4 score				*P*
	Low score		High score		
	negative (–)	weak (+)	moderate (++)	strong (+++)	
NCM	0	0	5	19	< 0.001
CA	3	5	9	7	
CRC	24	37	31	4	

**Table 4:** Correlation between pMKK4 score and clinicopathological characteristics of 96 CRC cases in the pilot-set

	pMKK4 score				*P*
	Low score		High score		
	negative (–)	weak (+)	moderate (++)	strong (+++)	
Gender					0.797
Male	13	22	17	3	
Female	11	15	14	1	
Age (years)					0.185
< 60	12	18	11	1	
≥ 60	12	19	20	3	
Tumor Differentiation					0.976
Poor	2	5	4	0	
Moderate	17	22	21	3	
Well	5	10	6	1	
KRAS mutation					0.237
Wild type	17	24	26	3	
Mutated	7	13	5	1	
Invasive Depth					0.106
T1	1	1	5	0	
T2	4	4	7	2	
T3	13	26	16	2	
T4	6	6	3	0	
LN metastasis					0.584
Absent	13	10	16	3	
Present	11	27	15	1	
Distant metastasis					0.017
Absent	16	34	28	4	
Present	8	3	3	0	
Liver metastasis					0.008
Absent	18	36	28	4	
Present	6	1	1	0	
TNM Stage					0.089
I	5	2	7	2	
II	6	9	7	1	
III	5	23	14	1	
IV	8	3	3	0	

### IHC staining of pMKK4 in NCM, CA, and CRC

**Figure 1:**
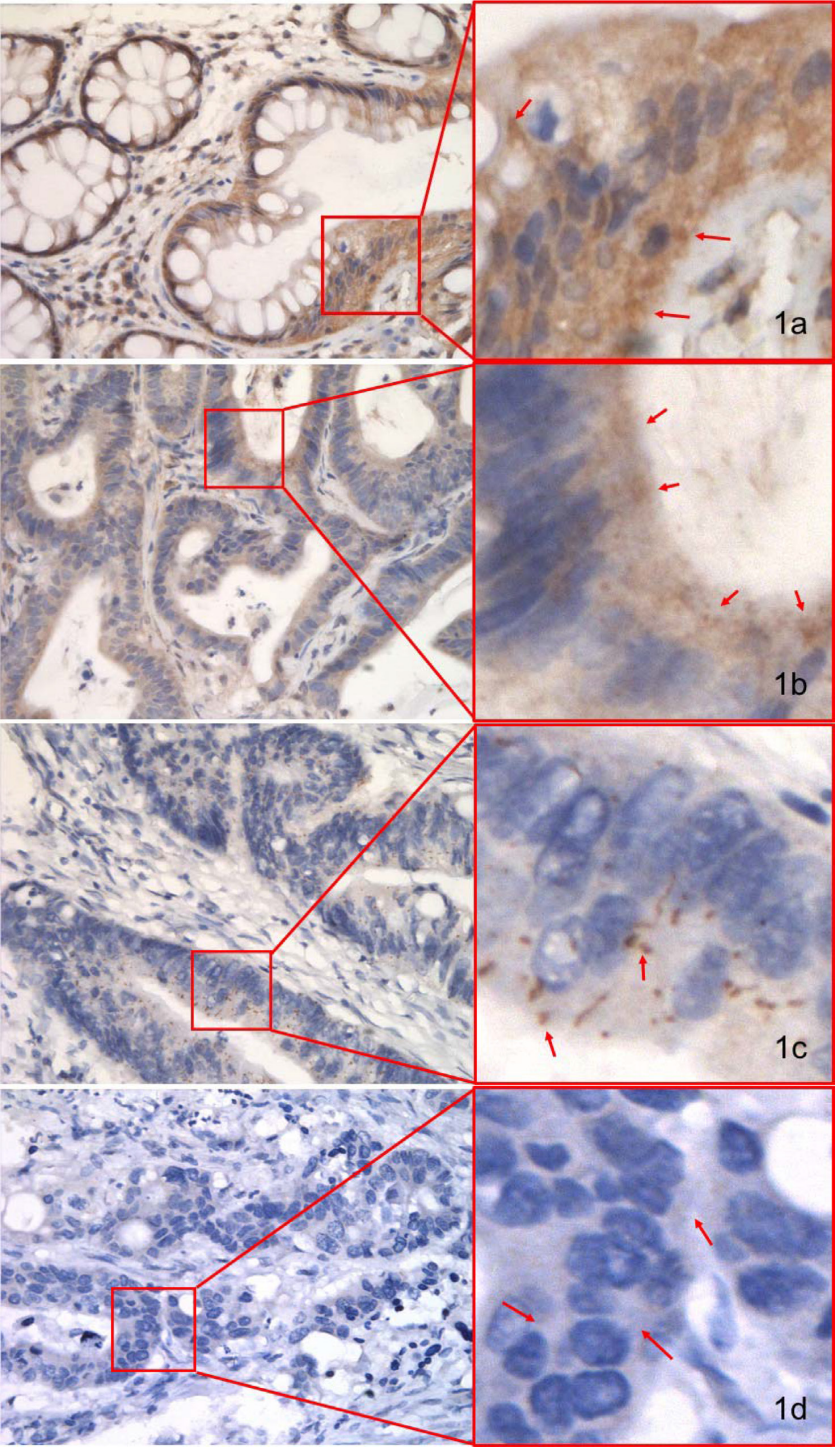
IHC staining of pMKK4 using selective immune-reactivity antibody with phosphopeptide containing S257/T261 site of MKK4 in normal colonic mucosa (NCM), colorectal adenoma (CA) and colorectal cancer (CRC) from the pilot set pMKK4 was cytoplasmic stained. Variable degree of pMKK4 expression level was showed in NCM (**A**, strong), CA (**B**, moderate) and CRC (**C**, weak) & (**D**, negative). Photos were captured by NIKKON microscope at 400×.

Cytoplasmic staining of pMKK4 was seen in 100.0% (24/24) of NCM, 87.5% (21/24) of CA, and 75.0% (72/96) of CRC (Table 3) pilot set specimens. All malignant cases displayed one of two distinct staining patterns; the first was similar to the pattern seen in benign tissue and was characterized by predominantly cytoplasmic staining, while the second was characterized by focal, weakly positive staining. All NCM samples showed moderate-to-strong pMKK4 expression mainly in the cytoplasm of glandular epithelial cells (Figure 1A). The majority of CA samples showed moderate pMKK4 staining (Figure 1B), while the majority of CRC specimens were negative for or showed weak pMKK4 staining (Figure 1C and 1D). In the pilot set, loss of pMKK4 expression was detected in 3 of 24 CA and 24 of 96 CRC samples. pMKK4 staining scores progressively increased in NCM more than in CA and CRC (*P* = 0.0035, 0.0002, respectively) (Table 3). In the validation set, 192 (43.8%), 128 (29.2%), 86 (19.6%), and 32 (7.3%) samples were labeled as negative, weak, moderate, and strong, respectively, for pMKK4 staining. Because none of the pilot set NCM samples were negative for or had weak pMKK4 staining, and because tumors with weak pMKK4 expression shared more biological similarities with pMKK4-negative tumors, validation set patients were then divided into “low pMKK4 score” (negative/weak) and “high pMKK4 score” (moderate/strong) groups for subsequent survival analysis (Table 2).

### Associations between pMKK4 and clinicopathological characteristics in CRC

In the pilot set, reduced pMKK4 expression was associated only with distant metastasis (*P* = 0.017), and especially liver metastasis (*P* = 0.008) (Table 3). In the validation set, however, downregulation of pMKK4 was associated with not only distant metastasis (*P* < 0.001), including liver metastasis (*P* < 0.001), but also with invasive depth (*P* < 0.001) and lymph node invasion (*P* < 0.001), which contributed to the direct association between pMKK4 expression and TNM stage (*P* < 0.001). Notably, pMKK4 was not significantly associated with tumor differentiation (*P* = 0.153).

### Univariate analysis for disease-free survival (DFS) and overall survival (OS)

**Figure 2:**
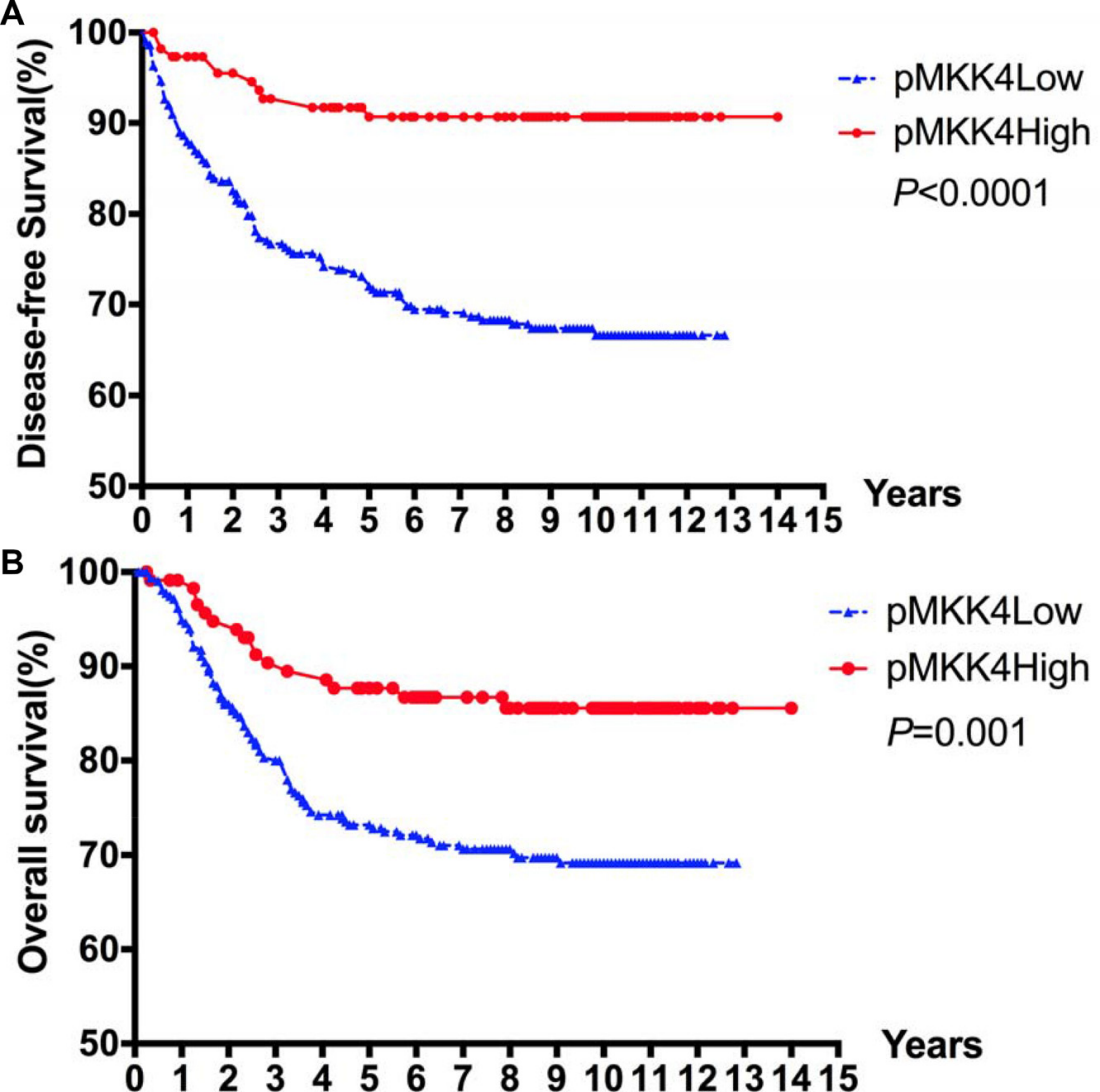
Kaplan–Meier survival curves for DFS (**A**) and OS (**B**) according to pMKK4 High score and Low score of patients with CRC. The 1, 5 and 10-year DFS rates in pMKK4 high score group and low score group were 97.4% VS. 88.0%, 90.7% VS. 71.3%, and 90.7% VS. 66.6%, accordingly. The 1, 5 and 10-year OS rates in pMKK4 high score group and low score group were 98.3% VS. 94.9%, 86.7% VS. 72.8%, and 85.6% VS. 71.8%, accordingly. The pMKK4 high score group showed a significant better DFS and OS than those of the pMKK4 low score group.

The mean survival times for the high and low pMKK4 score groups were 98.4 ± 42.3 and 82.4 ± 45.6 months, respectively. The 1-, 5-, and 10-year DFS rates in the high and low pMKK4 score groups were 97.4% versus 88.0%, 90.7% versus 71.3%, and 90.7% versus 66.6%, respectively. The 1-, 5-, and 10-year overall survival rates in the high and low pMKK4 score groups were 98.3% versus 94.9%, 86.7% versus 72.8%, and 85.6% versus 71.8%, respectively. Both DFS and OS were higher in the pMKK4 high score group than in the low score group (Figure 2). Univariate analysis using the Kaplan-Meier method indicated that pMKK4 score correlated with both DFS (HR = 0.3788, *P* < 0.001) and OS (HR = 0.4984, *P* = 0.001) (Table 5). In addition, tumor differentiation and TNM stage were also correlated with DFS and OS (Table 6).

**Table 5:** Univariate analysis of pMKK4 score and clinicopathological characteristics in the validation-set

	pMKK4 score				*P*
	Low score		High score		
	negative (–)	weak (+)	moderate (++)	strong (+++)	
Gender					0.361
Male	95	70	44	21	
Female	97	58	42	11	
Age (years)					0.573
< 60	95	57	36	13	
≥ 60	97	71	50	19	
Tumor Differentiation					0.153
Poof	30	18	8	2	
Moderate	147	102	66	28	
Well	15	8	12	2	
Invasive Depth					< 0.001
T1	1	1	8	1	
T2	23	18	18	12	
T3	142	92	48	19	
T4	26	17	12	0	
LN metastasis					< 0.001
Absent	92	64	60	28	
Present	100	64	26	4	
Distant metastasis(synchronous & metachronous)					
Absent	120	87	72	32	< 0.001
Present	72	41	14	0	
Liver metastasis(synchronous & metachronous)					< 0.001
Absent	124	90	75	32	
Present	68	38	11	0	
TNM Stage					< 0.001
I	19	12	18	13	
II	63	51	40	15	
III	76	58	23	4	
IV	34	7	5	0	

(*N* = 438) features.

**Table 6:** Univariate Cox analysis of potential prognostic factors influencing DFS and OS

	DFS			OS		
	Hazard Ratio	95% CI	*P*	Hazard Ratio	95% CI	*P*
Gender	1.264	0.861, 1.727	0.264	1.173	0.805, 1.711	0.406
Age (< 60 VS. ≥ 60)	1.067	0.752, 1.515	0.715	1.130	0.772, 1.655	0.503
Tumor Differentiation	1.706	1.175, 2.477	0.005	1.768	1.184, 2.641	0.005
pMKK4 Score	0.379	0.249, 0.577	< 0.001	0.498	0.328, 0.756	0.001
TNM Stage	3.807	2.912, 4.976	< 0.001	4.871	3.660, 6.483	< 0.001

### Multivariate Cox regression analysis of the predictive value of pMKK4 for DFS and OS

In multivariate analysis, the pMKK4 expression was also predictive of DFS (HR = 0.521, *P* = 0.024), but not of OS (HR = 0.791, *P* = 0.395), after adjusting for tumor differentiation and TNM stage. TNM stage remained the strongest independent predictor of CRC prognosis (Table 7).

**Table 7:** Multivariate Cox Analysis of pMKK4 score for DFS and OS after adjusting for tumor differentiation, pMKK4 score and TNM Stage in CRC

	DFS			OS		
	Hazard Ratio	95% CI	*P*	Hazard Ratio	95% CI	*P*
Tumor Differentiation	1.528	1.078, 2.165	0.017	1.576	1.094, 2.272	0.015
pMKK4 Score	0.521	0.296, 0.917	0.024	0.791	0.462, 1.375	0.395
TNM Stage	3.476	2.656, 4.549	< 0.001	4.614	3.462, 6.151	< 0.001

## DISCUSSION

Our results indicate that pMKK4 expression was downregulated in CRC. In previous studies, which focused on constitutive expression of MKK4 in various tumors, pMKK4 expression was rarely examined. Dual phosphorylation of MKK4 (pMKK4) at S257/T261 enhances the MKK4-induced upregulation of JNK/p38, which are involved in the regulation of apoptosis in various cells. It is therefore possible that pMKK4 expression may be a more accurate indicator of the functional status of MKK4 in tumor cells than MKK4 expression. In the current study, we examined associations between pMKK4 expression and clinicopathological factors in NCM, CA, and CRC. pMKK4 expression was highest in NCM and was reduced in CRC compared to both CA (*P* = 0.0035) and NCM (*P* = 0.0002), indicating that pMKK4 might play a tumor-suppressive role during CRC development. Despite the small sample size of the pilot set, decreased pMKK4 expression was still found to be associated with distant metastasis, especially liver metastasis; the same results were obtained in the validation set. These findings are in accordance with previous studies of MKK4, which has been identified as a suppressor of tumorigenesis and metastasis [3, 5, 6, 10].

MKK4 acts as a bottleneck in the SAPK signaling pathway. Most studies classify the MKK4 gene as a candidate tumor-suppressor gene and report that its expression is decreased in various tumors [5, 6, 11]. In contrast, Wang *et al*. [12] suggested that MKK4 has pro-oncogenic activity, but their interpretation of the findings is debatable. Cuenda pointed out that inhibition of MKK4 might have a paradoxical effect; instead of inhibiting proliferation, MKK4 inactivation might promote cancer cell survival by inhibiting JNK-mediated apoptosis pathways [8]. Furthermore, metastasis suppressors, such as NM23, MKK4, and KAI1, are not generally mutated, but rather “turned off” [13]. The regulation of MKK4 may also be tissue and site-specific [14]. Thus, the conflicting biological effects of MKK4 in human cancer might reflect the complexity of MAPK signal transduction.

Here, we report for the first time that pMKK4 expression is strongly correlated with pathological T stage (pT, invasive depth) in CRC. These correlations have not been examined in previous studies of MKK4, most of which found that MKK4 expression is negatively correlated with pT stage progression in invasive carcinoma [15]. In this study, pMKK4 expression was again correlated with pT stage, and lower pMKK4 expression was also associated with increases in locally advanced CRC and distant metastasis. However, pMKK4 expression was not associated with invasive depth or LN metastasis in the pilot set, likely due to the small case number. The JNK/p38 pathways, which are downstream of MKK4, play well-established roles in the regulation of cellular apoptosis, differentiation, and proliferation [9, 16, 17]. Reduced pMKK4 expression might therefore result in the downregulation of JNK and p38 and thereby promote tumor progression. Moreover, RAS-dependent signaling pathways, the activities of which are also correlated with sustained pMKK4 levels, are known to play an important role in apoptosis [12]. Taken together, our results and those of previous studies indicate that the pMKK4 protein suppresses CRC progression. Downregulation of pMKK4 might promote the proliferation of and inhibit apoptosis in CRC cells, ultimately leading to worse prognoses.

Our findings suggest that downregulation of pMKK4 expression is strongly associated with unfavorable tumor phenotypes and worse DFS in CRC. Local tumor invasion and distant metastasis, especially liver metastasis, were increased in patients with low pMKK4 expression (Tables 3 and 5). In the validation set, long-term follow-up data confirmed that DFS and OS were worse in patients with low pMKK4 scores than in those with high scores (Figure 2). More than half of the patients included in this study were in the early stages of CRC and underwent surgery without any preoperational new adjuvant therapies, which minimized the influence of those therapies on pMKK4 downregulation-induced effects on DFS. Multivariate analysis confirmed that pMKK4 was an independent risk factor for DFS, even after adjusting for tumor differentiation and TNM stage. Although pMKK4 expression was not an independent risk factor for OS, some postoperative treatment strategies (e.g., reoperation, regional ablation, hepatectomy, and adjuvant chemotherapy) might help to increase disease-free rates among patients with resectable recurrences. Although our findings suggest that pMKK4 expression may influence OS, TNM stage remained the strongest independent predictor of prognosis.

Some limitations of our study should be considered when interpreting the results. First, pMKK4 expression was evaluated using only tissue arrays; currently, there is no reliable model for testing pMKK4 expression in NCM, CA, or early-stage CRC samples either *in vitro* or *ex vivo*. Furthermore, NCM tissue is difficult to maintain in a culture system, and therefore is not readily available [13]. Second, different tumor cell line types and experimental models have previously produced paradoxical findings regarding pMKK4's effects. Moreover, most available colorectal epithelial cell lines have been derived from malignant tumors, and normal colorectal cell lines are rarely available. For these reasons, the specific biological mechanism of pMKK4 in NCM, CA, and CRC should be explored in the future works.

In summary, pMKK4 might function as a tumor suppressor in CRC. Downregulation of pMKK4 was associated with a more aggressive phenotype and with increases in local invasion and metastasis. pMKK4 was also strongly associated with DFS, implying that CRC patients with low pMKK4 expression should be monitored carefully for the early detection of possible recurrences, especially liver metastasis.

## MATERIALS AND METHODS

### Patients and specimens

The pilot set consisted of formalin-fixed and paraffin-embedded specimens from 24 CA and 96 CRC cases collected during initial surgical resections between March 2008 and August 2009 that were randomly selected from the specimen bank of the Sixth Affiliated Hospital, Sun Yat-sen University. Twenty-four NCM specimens were obtained for comparison during procedures for prolapse and hemorrhoids. Clinicopathological characteristics are summarized in Table 1.

The validation set consisted of 438 cases randomly selected from a group of 2000 CRC patients who had not received any preoperative treatments and who underwent initial surgical resections between January 2000 and December 2006 at the First Affiliated Hospital, Sun Yat-sen University. Microarray data were available for all patients, and clinicopathological characteristics are summarized in Table 2. The median duration of follow-up was 102.0 months (± 45.2 months). The use of patient specimens was approved by the local ethics committees of both the First Affiliated Hospital and the Sixth Affiliated Hospital of Sun Yat-sen University.

### Tissue microarray

A MiniCore^®^ tissue arrayer (Alphelys, France) was used to construct the tissue microarray [18]. Hematoxylin and eosin-stained slides of all formalin-fixed paraffin-embedded specimens were examined by the experienced pathologists Huang Y and Fan XJ, who selected areas containing CRC, CA, or NCM while avoiding areas of necrosis, inflammation, and keratinization. To assess reproducibility, we selected two cores from different areas of a single specimen. The tissue microarray construction procedure used here has been described elsewhere [18]. Briefly, tissue cylinders 1.0 mm in diameter were punched from representative tissue areas and embedded in single paraffin blocks (2.6 cm × 2.2 cm). Serial sections (4.0 μm) were cut from these blocks with a microtome and mounted on APES-coated slides for IHC analysis.

### IHC staining

An affinity-purified rabbit anti-phospho-MKK4 polyclonal antibody (Catalog Number, AF2990) showing selective immunoreactivity with a phosphopeptide containing the S257/T261 site of MKK4 was obtained from R&D, Inc. (USA). The antibody was employed for subsequent IHC assays at a dilution of 1:100. Standard indirect immuno-peroxidase procedures were used for IHC analysis. Slides were de-waxed and rehydrated in dH_2_O. Following microwave-mediated antigen retrieval in 0.01 M sodium citrate (pH = 6.0), endogenous peroxidase activity was blocked using 3% H_2_O_2_ for 10 min, and the sections were incubated with 10% normal goat serum for 30 min at room temperature. Primary antibody was then added to the slides, which were then incubated overnight at 4°C and washed. Subsequently, the sections were incubated with HRP-conjugated secondary antibody (Rabbit/Mouse, Peroxidase/DAB+, Code K5007, DAKO REAL^™^ EnVision^™^ Detection System) for 30 min at room temperature. For visualization of the antigen, sections were stained with DAB+ chromogen for 30 s according to the manufacturer's instructions and counterstained with Gill's hematoxylin. The primary antibody was omitted as a negative control.

### pMKK4 staining score

Two investigators (pathologists Dr. Huang Y and Dr. Fan XJ) independently evaluated the IHC staining. The results were evaluated by assigning IHC scores as previously described. The mean percentage of positive tumor cells in at least five areas under 400× magnification was determined and assigned to one of five categories: < 5%, 0; 5–25%, 1; 25–50%, 2; 50–75%, 3; and > 75%, 4. The intensity of pMKK4 immunostaining was scored as follows: negative, 0; weak, 1; moderate, 2; and intense, 3. The IHC score was calculated by adding the mean percentage and intensity values and was assigned to one of four categories: 0–1, negative (–); 2–3, weak (+); 4–5, moderate (++); and 6–7, strong (+++) [19]. Specimens with IHC scores 0–3 (–/+) were assigned to the low pMKK4 score group, while specimens with IHC scores 4–7 (++/+++) were assigned to the high score group. In cases for which the observers provided similar scores, the two scores were averaged. In the small subset of cases in which there were significant differences in initial interpretations, scores were assigned by consensus.

### Statistical analysis

The Kruskal-Wallis and Mann-Whitney tests were used to determine differences between two groups and among more than two groups, respectively. *P* < 0.05 was considered statistically significant. Univariate survival analysis for pMKK4 was performed using the Kaplan-Meier method. Univariate analyses for gender, age, tumor differentiation, TNM stage, and pMKK4 score were performed using Cox proportional hazards regression. Hazard ratios and 95% confidence intervals were obtained. Multivariate survival analysis was performed using all significant variables (*P* < 0.05) from the univariate analysis. Analyses were performed using IBM SPSS 23.0 and Graphpad Prism 6.
